# Lamotriginium dihydrogen phosphate–4-(dimethyl­amino)­benzaldehyde (1/1)

**DOI:** 10.1107/S1600536810034884

**Published:** 2010-09-15

**Authors:** Syed Naeem Razzaq, Islam Ullah Khan, Onur Şahin, Orhan Büyükgüngör

**Affiliations:** a Materials Chemistry Laboratry, Department of Chemistry, GC University, Lahore 54000, Pakistan; b Department of Physics, Ondokuz Mayıs University, TR-55139 Samsun, Turkey

## Abstract

In the title compound, C_9_H_8_Cl_2_N_5_^+^·H_2_PO_4_^−^·C_9_H_11_NO [systematic name: 3,5-diamino-6-(2,3-dichloro­phen­yl)-1,2,4-triazin-2-ium dihydrogen phosphate–4-(dimethyl­amino)­benz­alde­hyde (1/1)], inter­molecular N—H⋯O and O—H⋯O hydrogen bonds produce *R*_2_^2^(8) and *R*_3_^2^(8) rings, generating a layer. Inter­molecular N—H⋯N inter­actions also occur. The dihedral angle between the rings in the cation is 71.73 (12)°.

## Related literature

For the graph-set analysis of hydrogen-bond patterns, see: Bernstein *et al.* (1995 ▶). For related structures, see: Sridhar & Ravikumar (2006 ▶). For bond-valence calculations, see: Brese & O’Keeffe (1991 ▶). 
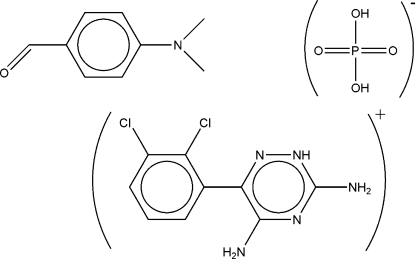


## Experimental

### 

#### Crystal data

C_9_H_8_Cl_2_N_5_^+^·H_2_PO_4_^−^·C_9_H_11_NO
*M* *_r_* = 503.28
Triclinic, 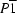

*a* = 8.1586 (4) Å
*b* = 10.5206 (6) Å
*c* = 13.6359 (7) Å
α = 98.665 (3)°
β = 98.131 (4)°
γ = 99.746 (3)°
*V* = 1123.49 (10) Å^3^
*Z* = 2
Mo *K*α radiation
μ = 0.40 mm^−1^
*T* = 296 K
0.31 × 0.27 × 0.25 mm

#### Data collection

Bruker Kappa APEXII CCD area detector diffractometer
19715 measured reflections
4310 independent reflections
3219 reflections with *I* > 2σ(*I*)
*R*_int_ = 0.031

#### Refinement

*R*[*F* ^2^ > 2σ(*F* ^2^)] = 0.068
*wR*(*F* ^2^) = 0.240
*S* = 1.07
4310 reflections
315 parameters
8 restraints
H atoms treated by a mixture of independent and constrained refinement
Δρ_max_ = 1.82 e Å^−3^
Δρ_min_ = −0.54 e Å^−3^

### 

Data collection: *APEX2* (Bruker, 2005 ▶); cell refinement: *SAINT* (Bruker, 2002 ▶); data reduction: *SAINT*; program(s) used to solve structure: *SHELXS97* (Sheldrick, 2008 ▶); program(s) used to refine structure: *SHELXL97* (Sheldrick, 2008 ▶); molecular graphics: *ORTEP-3 for Windows* (Farrugia, 1997 ▶); software used to prepare material for publication: *WinGX* (Farrugia, 1999 ▶).

## Supplementary Material

Crystal structure: contains datablocks global, I. DOI: 10.1107/S1600536810034884/jh2198sup1.cif

Structure factors: contains datablocks I. DOI: 10.1107/S1600536810034884/jh2198Isup2.hkl

Additional supplementary materials:  crystallographic information; 3D view; checkCIF report

## Figures and Tables

**Table 1:** Hydrogen-bond geometry (Å, °)

*D*—H⋯*A*	*D*—H	H⋯*A*	*D*⋯*A*	*D*—H⋯*A*
O1—H1⋯O2^i^	0.82 (6)	1.82 (5)	2.627 (5)	171 (7)
O3—H3⋯O4^ii^	0.81 (2)	1.80 (2)	2.602 (4)	169 (7)
N4—H4*A*⋯O5^iii^	0.87 (4)	2.08 (3)	2.888 (5)	155 (5)
N4—H4*B*⋯O4	0.86 (2)	1.86 (2)	2.719 (4)	177 (5)
N5—H5*A*⋯N3^iv^	0.89 (5)	2.21 (5)	3.088 (5)	171 (6)
N5—H5*B*⋯O5^v^	0.87 (2)	2.14 (5)	2.799 (5)	132 (5)
N2—H2⋯O2	0.87 (2)	1.81 (2)	2.663 (4)	170 (6)

Symmetry codes: (i) 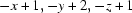; (ii) 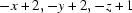; (iii) 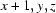; (iv) 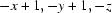; (v) 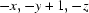.

**Fig. 1.**
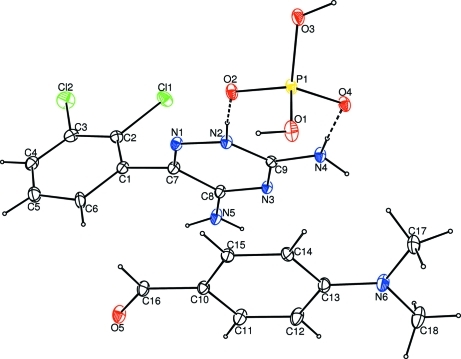
A view of one molecule of (I), showing the atom-numbering scheme. Displacement ellipsoids are drawn at the 30% probability level and H atoms are shown as small spheres of arbitrary radii. Hydrogen bonds are indicated by dashed lines.

**Fig. 2.**
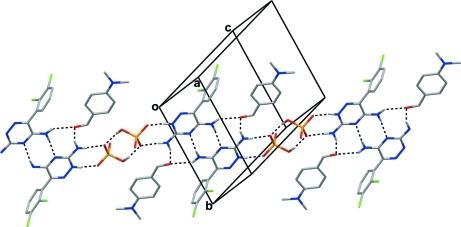
Part of the crystal structure of (I), showing the formation of a hydrogen-bonded sheet built from R22(8) and R32(8) rings. For the sake of clarity, H atoms not involved in the motif shown have been omitted.

## supplementary crystallographic information

### Comment

The title compound is a salt of Lamotrigine (Sridhar *et al.*, 2006) an anticonvulsant drug used in the treatment of epilepsy and bipolar disorder. Herein we report the synthesis and crystal structure of title compound (I).

The molecular structure and atom-labelling scheme are shown in Fig. 1. Selected bond distances and angles are given in Table 1. The P1—O2 and P1—O4 bond lengths [1.506 (3)Å and 1.496 (3) Å] indicate significant single-bond character, whereas the P1—O1 and P1—O3 bond lengths [1.567 (4)Å and 1.559 (3) Å] are indicative of significant double-bond character. The O—P—O angles lie in the range 107.15 (19)–114.31 (16)°. Linkages P1—O1 and P1—O3 constitute POH groups, as confirmed both by the location of H atoms in the difference Fourier maps and by bond-valence calculations (Brese & O'Keeffe, 1991).

The amino atom N5 in the molecule at (*x*, *y*, *z*) acts as a hydrogen-bond donor (Table 2) to atom N3^iv^ so forming a centrosymmetric *R*_2_^2^(8) ring (Bernstein *et al.*, 1995) centred at (1/2, 1/2, 0). Similarly, atom O3 in the molecule at (*x*, *y*, *z*) acts as a hydrogen-bond donor to atom O4^ii^ so forming a centrosymmetric *R*_2_^2^(8) ring centred at (1, 1, 1/2). The combination of N—H···O and O—H···O hydrogen bonds generates *R*_3_^2^(8) and *R*_2_^2^(8) rings parallel to the [111] direction (Fig. 2).

### Experimental

15 ml (0.06*M*) Methanolic solution of Lamotrigine is mixed with 15 ml (0.06*M*) Methanolic solution of 4-dimethylaminobenzaldehyde in glass beaker on hot plate stirrer for 10 minutes. Then add 3–4 drops of (85%) phosphoric acid and again mix for 4–5 h on hot plate stirrer. Yellow prisms of (I) were obtained by slow evaporation from methanol.

### Refinement

All H atoms bound to C atoms were refined using a riding model, with C—H = 0.93Å and *U*_iso_(H) = 1.2*U*_eq_(C) for aromatic C atoms and C—H = 0.96Å and *U*_iso_(H) = 1.5*U*_eq_(C) for methyl C atoms. Other H atoms bound to N and O atoms were located in difference maps and refined subject to a *DFIX* restraint of O—H = 0.82 (2)Å and N—H = 0.87 (2) Å.

### Crystal data

**Table:**

C_9_H_8_Cl_2_N_5_^+^·H_2_PO_4_^−^·C_9_H_11_NO	*Z* = 2
*M**_r_* = 503.28	*F*(000) = 520
Triclinic, *P*1	*D*_x_ = 1.488 Mg m^−^^3^
Hall symbol: -P 1	Mo *K*α radiation, λ = 0.71073 Å
*a* = 8.1586 (4) Å	Cell parameters from 8437 reflections
*b* = 10.5206 (6) Å	θ = 2.3–28.0°
*c* = 13.6359 (7) Å	µ = 0.40 mm^−^^1^
α = 98.665 (3)°	*T* = 296 K
β = 98.131 (4)°	Prism, yellow
γ = 99.746 (3)°	0.31 × 0.27 × 0.25 mm
*V* = 1123.49 (10) Å^3^	

### Data collection

**Table:**

Bruker Kappa APEXII CCD area detector diffractometer	3219 reflections with *I* > 2σ(*I*)
Radiation source: fine-focus sealed tube	*R*_int_ = 0.031
graphite	θ_max_ = 26.0°, θ_min_ = 1.5°
phi and ω scans	*h* = −10→9
19715 measured reflections	*k* = −12→12
4310 independent reflections	*l* = −16→16

### Refinement

**Table:**

Refinement on *F*^2^	Primary atom site location: structure-invariant direct methods
Least-squares matrix: full	Secondary atom site location: difference Fourier map
*R*[*F*^2^ > 2σ(*F*^2^)] = 0.068	Hydrogen site location: inferred from neighbouring sites
*wR*(*F*^2^) = 0.240	H atoms treated by a mixture of independent and constrained refinement
*S* = 1.07	*w* = 1/[σ^2^(*F*_o_^2^) + (0.1269*P*)^2^ + 2.426*P*] where *P* = (*F*_o_^2^ + 2*F*_c_^2^)/3
4310 reflections	(Δ/σ)_max_ = 0.011
315 parameters	Δρ_max_ = 1.82 e Å^−^^3^
8 restraints	Δρ_min_ = −0.54 e Å^−^^3^

### Special details

**Table:**

Geometry. All e.s.d.'s (except the e.s.d. in the dihedral angle between two l.s. planes) are estimated using the full covariance matrix. The cell e.s.d.'s are taken into account individually in the estimation of e.s.d.'s in distances, angles and torsion angles; correlations between e.s.d.'s in cell parameters are only used when they are defined by crystal symmetry. An approximate (isotropic) treatment of cell e.s.d.'s is used for estimating e.s.d.'s involving l.s. planes.
Refinement. Refinement of *F*^2^ against ALL reflections. The weighted *R*-factor *wR* and goodness of fit *S* are based on *F*^2^, conventional *R*-factors *R* are based on *F*, with *F* set to zero for negative *F*^2^. The threshold expression of *F*^2^ > σ(*F*^2^) is used only for calculating *R*-factors(gt) *etc*. and is not relevant to the choice of reflections for refinement. *R*-factors based on *F*^2^ are statistically about twice as large as those based on *F*, and *R*- factors based on ALL data will be even larger.

### Fractional atomic coordinates and isotropic or equivalent isotropic displacement parameters (Å^2^)

**Table:**

	*x*	*y*	*z*	*U*_iso_*/*U*_eq_	
C1	0.1434 (6)	0.8025 (4)	0.0052 (3)	0.0389 (10)	
C2	0.1627 (6)	0.8530 (4)	−0.0804 (3)	0.0431 (10)	
C3	0.0246 (7)	0.8825 (5)	−0.1405 (4)	0.0511 (12)	
C4	−0.1267 (7)	0.8634 (5)	−0.1102 (4)	0.0529 (13)	
H4	−0.2188	0.8840	−0.1487	0.064*	
C5	−0.1487 (7)	0.8146 (6)	−0.0248 (4)	0.0577 (13)	
H5	−0.2550	0.8049	−0.0065	0.069*	
C6	−0.0185 (6)	0.7787 (5)	0.0361 (5)	0.0570 (14)	
H6	−0.0353	0.7419	0.0926	0.068*	
C7	0.2925 (6)	0.7763 (4)	0.0699 (3)	0.0378 (9)	
C8	0.3738 (5)	0.6668 (4)	0.0404 (3)	0.0372 (9)	
C9	0.5728 (5)	0.7406 (4)	0.1818 (3)	0.0301 (8)	
C10	0.2160 (5)	0.5795 (4)	0.2953 (3)	0.0366 (9)	
C11	0.2531 (7)	0.4619 (5)	0.2513 (4)	0.0506 (12)	
H11	0.1751	0.4067	0.1995	0.061*	
C12	0.4023 (7)	0.4262 (5)	0.2830 (4)	0.0578 (14)	
H12	0.4235	0.3466	0.2530	0.069*	
C13	0.5258 (6)	0.5084 (5)	0.3607 (3)	0.0421 (10)	
C14	0.4861 (5)	0.6265 (4)	0.4056 (3)	0.0388 (10)	
H14	0.5626	0.6817	0.4581	0.047*	
C15	0.3367 (5)	0.6605 (4)	0.3728 (3)	0.0380 (9)	
H15	0.3142	0.7397	0.4027	0.046*	
C16	0.0600 (6)	0.6187 (4)	0.2665 (3)	0.0416 (10)	
H16	0.0468	0.6993	0.2997	0.050*	
C17	0.8050 (7)	0.5653 (6)	0.4645 (5)	0.0649 (15)	
H17A	0.7933	0.6538	0.4610	0.097*	
H17B	0.9142	0.5534	0.4510	0.097*	
H17C	0.7933	0.5480	0.5306	0.097*	
C18	0.7119 (9)	0.3496 (7)	0.3527 (6)	0.080 (2)	
H18A	0.6212	0.2815	0.3586	0.120*	
H18B	0.8149	0.3387	0.3913	0.120*	
H18C	0.7238	0.3447	0.2833	0.120*	
N1	0.3508 (5)	0.8558 (3)	0.1537 (3)	0.0387 (8)	
N2	0.4890 (4)	0.8348 (3)	0.2110 (2)	0.0348 (8)	
H2	0.525 (7)	0.894 (4)	0.265 (3)	0.062 (17)*	
N3	0.5143 (4)	0.6533 (3)	0.0951 (2)	0.0351 (8)	
N4	0.7119 (5)	0.7329 (4)	0.2380 (3)	0.0386 (8)	
H4A	0.752 (6)	0.662 (3)	0.225 (4)	0.046*	
H4B	0.755 (6)	0.784 (4)	0.294 (2)	0.046*	
N5	0.3095 (6)	0.5793 (4)	−0.0417 (3)	0.0549 (12)	
H5A	0.358 (7)	0.514 (4)	−0.064 (4)	0.066*	
H5B	0.207 (4)	0.574 (6)	−0.073 (4)	0.066*	
N6	0.6754 (6)	0.4756 (4)	0.3905 (4)	0.0564 (11)	
Cl1	0.36141 (18)	0.88738 (17)	−0.11201 (11)	0.0679 (5)	
Cl2	0.0485 (3)	0.9446 (2)	−0.24770 (12)	0.0872 (6)	
P1	0.73789 (13)	0.97774 (10)	0.46306 (8)	0.0335 (3)	
O1	0.6763 (4)	0.9254 (4)	0.5559 (2)	0.0525 (9)	
H1	0.600 (6)	0.955 (6)	0.578 (5)	0.07 (2)*	
O2	0.5893 (4)	0.9922 (3)	0.3893 (2)	0.0387 (7)	
O3	0.8488 (4)	1.1173 (3)	0.5014 (3)	0.0515 (9)	
H3	0.949 (3)	1.122 (6)	0.521 (5)	0.07 (2)*	
O4	0.8423 (3)	0.8862 (3)	0.4206 (2)	0.0380 (7)	
O5	−0.0585 (4)	0.5567 (3)	0.2023 (3)	0.0540 (9)	

### Atomic displacement parameters (Å^2^)

**Table:**

	*U*^11^	*U*^22^	*U*^33^	*U*^12^	*U*^13^	*U*^23^
C1	0.042 (2)	0.035 (2)	0.036 (2)	0.0089 (17)	−0.0034 (18)	0.0011 (17)
C2	0.043 (3)	0.043 (2)	0.038 (2)	0.0045 (19)	0.0011 (19)	−0.0010 (19)
C3	0.061 (3)	0.044 (3)	0.042 (3)	0.007 (2)	−0.008 (2)	0.006 (2)
C4	0.055 (3)	0.046 (3)	0.052 (3)	0.013 (2)	−0.009 (2)	0.003 (2)
C5	0.043 (3)	0.066 (3)	0.065 (3)	0.016 (2)	0.005 (2)	0.012 (3)
C6	0.032 (2)	0.060 (3)	0.073 (4)	0.018 (2)	−0.003 (2)	−0.005 (3)
C7	0.042 (2)	0.036 (2)	0.032 (2)	0.0090 (18)	−0.0045 (17)	0.0000 (16)
C8	0.038 (2)	0.036 (2)	0.033 (2)	0.0109 (17)	−0.0067 (17)	−0.0009 (16)
C9	0.029 (2)	0.033 (2)	0.0252 (18)	0.0041 (15)	0.0014 (15)	0.0032 (15)
C10	0.036 (2)	0.036 (2)	0.035 (2)	0.0088 (17)	−0.0029 (17)	0.0038 (17)
C11	0.053 (3)	0.041 (2)	0.048 (3)	0.013 (2)	−0.013 (2)	−0.005 (2)
C12	0.061 (3)	0.044 (3)	0.064 (3)	0.025 (2)	−0.006 (3)	−0.006 (2)
C13	0.037 (2)	0.049 (3)	0.043 (2)	0.0137 (19)	0.0019 (18)	0.012 (2)
C14	0.031 (2)	0.040 (2)	0.041 (2)	0.0042 (17)	−0.0025 (17)	0.0046 (18)
C15	0.037 (2)	0.035 (2)	0.039 (2)	0.0071 (17)	0.0007 (17)	0.0016 (17)
C16	0.041 (2)	0.045 (2)	0.037 (2)	0.0151 (19)	−0.0046 (18)	0.0030 (19)
C17	0.041 (3)	0.080 (4)	0.076 (4)	0.020 (3)	−0.007 (3)	0.029 (3)
C18	0.075 (5)	0.080 (4)	0.094 (5)	0.051 (4)	0.005 (4)	0.015 (4)
N1	0.040 (2)	0.0405 (19)	0.0329 (18)	0.0127 (15)	−0.0031 (15)	0.0026 (15)
N2	0.0364 (19)	0.0372 (19)	0.0262 (17)	0.0101 (15)	−0.0037 (14)	−0.0035 (14)
N3	0.0362 (19)	0.0362 (18)	0.0286 (17)	0.0111 (14)	−0.0045 (14)	−0.0028 (14)
N4	0.036 (2)	0.045 (2)	0.0290 (17)	0.0139 (16)	−0.0065 (14)	−0.0063 (15)
N5	0.056 (3)	0.049 (2)	0.046 (2)	0.023 (2)	−0.0238 (19)	−0.0178 (18)
N6	0.049 (3)	0.062 (3)	0.063 (3)	0.025 (2)	0.001 (2)	0.019 (2)
Cl1	0.0536 (8)	0.0924 (11)	0.0575 (8)	0.0018 (7)	0.0109 (6)	0.0257 (7)
Cl2	0.0991 (13)	0.1090 (14)	0.0548 (9)	0.0153 (10)	−0.0059 (8)	0.0430 (9)
P1	0.0261 (5)	0.0405 (6)	0.0291 (5)	0.0093 (4)	−0.0027 (4)	−0.0049 (4)
O1	0.042 (2)	0.083 (3)	0.0402 (18)	0.0265 (18)	0.0094 (15)	0.0162 (17)
O2	0.0304 (15)	0.0498 (17)	0.0313 (15)	0.0123 (12)	−0.0043 (11)	−0.0031 (12)
O3	0.0325 (18)	0.0419 (18)	0.068 (2)	0.0122 (14)	−0.0107 (15)	−0.0153 (15)
O4	0.0284 (15)	0.0406 (16)	0.0378 (15)	0.0082 (12)	−0.0023 (11)	−0.0094 (12)
O5	0.046 (2)	0.060 (2)	0.0463 (19)	0.0173 (16)	−0.0176 (15)	−0.0037 (16)

### Geometric parameters (Å, °)

**Table:**

C1—C2	1.371 (6)	C13—C14	1.409 (6)
C1—C6	1.436 (7)	C14—C15	1.362 (6)
C1—C7	1.489 (6)	C14—H14	0.9300
C2—C3	1.404 (7)	C15—H15	0.9300
C2—Cl1	1.730 (5)	C16—O5	1.225 (5)
C3—C4	1.349 (8)	C16—H16	0.9300
C3—Cl2	1.711 (5)	C17—N6	1.449 (7)
C4—C5	1.363 (8)	C17—H17A	0.9600
C4—H4	0.9300	C17—H17B	0.9600
C5—C6	1.393 (7)	C17—H17C	0.9600
C5—H5	0.9300	C18—N6	1.443 (7)
C6—H6	0.9300	C18—H18A	0.9600
C7—N1	1.286 (5)	C18—H18B	0.9600
C7—C8	1.458 (6)	C18—H18C	0.9600
C8—N5	1.317 (5)	N1—N2	1.347 (5)
C8—N3	1.318 (5)	N2—H2	0.87 (2)
C9—N4	1.298 (5)	N4—H4A	0.87 (4)
C9—N2	1.341 (5)	N4—H4B	0.857 (19)
C9—N3	1.353 (5)	N5—H5A	0.89 (5)
C10—C11	1.392 (6)	N5—H5B	0.87 (2)
C10—C15	1.401 (6)	P1—O4	1.496 (3)
C10—C16	1.424 (6)	P1—O2	1.506 (3)
C11—C12	1.366 (7)	P1—O3	1.559 (3)
C11—H11	0.9300	P1—O1	1.567 (4)
C12—C13	1.419 (7)	O1—H1	0.82 (6)
C12—H12	0.9300	O3—H3	0.81 (2)
C13—N6	1.348 (6)		
			
C2—C1—C6	120.9 (4)	C13—C14—H14	119.7
C2—C1—C7	120.1 (4)	C14—C15—C10	121.9 (4)
C6—C1—C7	118.9 (4)	C14—C15—H15	119.1
C1—C2—C3	120.9 (4)	C10—C15—H15	119.1
C1—C2—Cl1	119.6 (4)	O5—C16—C10	126.5 (4)
C3—C2—Cl1	119.4 (4)	O5—C16—H16	116.8
C4—C3—C2	118.2 (5)	C10—C16—H16	116.8
C4—C3—Cl2	120.9 (4)	N6—C17—H17A	109.5
C2—C3—Cl2	120.9 (4)	N6—C17—H17B	109.5
C3—C4—C5	121.9 (5)	H17A—C17—H17B	109.5
C3—C4—H4	119.0	N6—C17—H17C	109.5
C5—C4—H4	119.0	H17A—C17—H17C	109.5
C4—C5—C6	122.8 (5)	H17B—C17—H17C	109.5
C4—C5—H5	118.6	N6—C18—H18A	109.5
C6—C5—H5	118.6	N6—C18—H18B	109.5
C5—C6—C1	115.1 (5)	H18A—C18—H18B	109.5
C5—C6—H6	122.4	N6—C18—H18C	109.5
C1—C6—H6	122.4	H18A—C18—H18C	109.5
N1—C7—C8	120.0 (4)	H18B—C18—H18C	109.5
N1—C7—C1	117.6 (4)	C7—N1—N2	117.8 (3)
C8—C7—C1	122.3 (3)	C9—N2—N1	123.0 (3)
N5—C8—N3	118.3 (4)	C9—N2—H2	123 (4)
N5—C8—C7	121.0 (4)	N1—N2—H2	113 (4)
N3—C8—C7	120.7 (4)	C8—N3—C9	117.1 (3)
N4—C9—N2	119.3 (3)	C9—N4—H4A	118 (3)
N4—C9—N3	119.7 (4)	C9—N4—H4B	124 (4)
N2—C9—N3	121.1 (3)	H4A—N4—H4B	117 (5)
C11—C10—C15	118.0 (4)	C8—N5—H5A	124 (4)
C11—C10—C16	122.8 (4)	C8—N5—H5B	121 (4)
C15—C10—C16	119.2 (4)	H5A—N5—H5B	114 (6)
C12—C11—C10	121.0 (4)	C13—N6—C18	121.9 (5)
C12—C11—H11	119.5	C13—N6—C17	120.8 (4)
C10—C11—H11	119.5	C18—N6—C17	117.2 (5)
C11—C12—C13	121.3 (4)	O4—P1—O2	114.31 (16)
C11—C12—H12	119.4	O4—P1—O3	109.93 (18)
C13—C12—H12	119.4	O2—P1—O3	107.15 (19)
N6—C13—C14	121.1 (4)	O4—P1—O1	107.16 (19)
N6—C13—C12	121.6 (4)	O2—P1—O1	110.19 (18)
C14—C13—C12	117.3 (4)	O3—P1—O1	107.9 (2)
C15—C14—C13	120.6 (4)	P1—O1—H1	118 (5)
C15—C14—H14	119.7	P1—O3—H3	118 (5)
			
C6—C1—C2—C3	−0.1 (7)	C10—C11—C12—C13	−0.9 (9)
C7—C1—C2—C3	−177.8 (4)	C11—C12—C13—N6	−178.1 (5)
C6—C1—C2—Cl1	177.0 (4)	C11—C12—C13—C14	1.6 (8)
C7—C1—C2—Cl1	−0.6 (6)	N6—C13—C14—C15	177.9 (4)
C1—C2—C3—C4	1.8 (7)	C12—C13—C14—C15	−1.8 (7)
Cl1—C2—C3—C4	−175.4 (4)	C13—C14—C15—C10	1.2 (7)
C1—C2—C3—Cl2	−179.6 (3)	C11—C10—C15—C14	−0.4 (7)
Cl1—C2—C3—Cl2	3.2 (6)	C16—C10—C15—C14	178.1 (4)
C2—C3—C4—C5	−1.2 (7)	C11—C10—C16—O5	0.5 (8)
Cl2—C3—C4—C5	−179.7 (4)	C15—C10—C16—O5	−177.9 (5)
C3—C4—C5—C6	−1.3 (8)	C8—C7—N1—N2	0.9 (6)
C4—C5—C6—C1	2.9 (8)	C1—C7—N1—N2	−178.0 (4)
C2—C1—C6—C5	−2.1 (7)	N4—C9—N2—N1	174.9 (4)
C7—C1—C6—C5	175.6 (4)	N3—C9—N2—N1	−5.4 (6)
C2—C1—C7—N1	105.5 (5)	C7—N1—N2—C9	4.1 (6)
C6—C1—C7—N1	−72.2 (6)	N5—C8—N3—C9	−176.6 (4)
C2—C1—C7—C8	−73.4 (6)	C7—C8—N3—C9	3.7 (6)
C6—C1—C7—C8	108.9 (5)	N4—C9—N3—C8	−179.1 (4)
N1—C7—C8—N5	175.3 (5)	N2—C9—N3—C8	1.2 (6)
C1—C7—C8—N5	−5.8 (7)	C14—C13—N6—C18	173.7 (5)
N1—C7—C8—N3	−4.9 (7)	C12—C13—N6—C18	−6.7 (8)
C1—C7—C8—N3	173.9 (4)	C14—C13—N6—C17	−4.5 (7)
C15—C10—C11—C12	0.3 (8)	C12—C13—N6—C17	175.2 (5)
C16—C10—C11—C12	−178.2 (5)		

### Hydrogen-bond geometry (Å, °)

**Table:**

*D*—H···*A*	*D*—H	H···*A*	*D*···*A*	*D*—H···*A*
O1—H1···O2^i^	0.82 (6)	1.82 (5)	2.627 (5)	171 (7)
O3—H3···O4^ii^	0.81 (2)	1.80 (2)	2.602 (4)	169 (7)
N4—H4A···O5^iii^	0.87 (4)	2.08 (3)	2.888 (5)	155 (5)
N4—H4B···O4	0.86 (2)	1.86 (2)	2.719 (4)	177 (5)
N5—H5A···N3^iv^	0.89 (5)	2.21 (5)	3.088 (5)	171 (6)
N5—H5B···O5^v^	0.87 (2)	2.14 (5)	2.799 (5)	132 (5)
N2—H2···O2	0.87 (2)	1.81 (2)	2.663 (4)	170 (6)

Symmetry codes: (i) −*x*+1, −*y*+2, −*z*+1; (ii) −*x*+2, −*y*+2, −*z*+1; (iii) *x*+1, *y*, *z*; (iv) −*x*+1, −*y*+1, −*z*; (v) −*x*, −*y*+1, −*z*.
